# The Crawford W. Long Monument

**Published:** 1908-06

**Authors:** 


### THE CRAWFORD W. LONG MONUMENT.

It is particulaıly fitting that a monument be erected by the medical profession of Georgia to one of her most distinguished citizens, Crawford W. Long, the discoverer of ether anesthesia.

With this purpose in view the Athens Woman's Club has undertaken the collection of funds for the erection of this memorial and it is earnestly desired that every physician in the state make a prompt contribution and thereby assist in honoring a notable member of the profession—the discoverer of one of the most useful of anesthetics.

It is a sad commentary and a reflection upon Georgia that Long should have received greater honors in other countries than he has in his own State. A prompt contribution and hearty co-operation of the physicians of this State will enable the Woman's Club to erect a monument in Athens, the home of this distinguished citizen, and thus in a measure may we neutralize our past neglect and delinquency. The United States has honored the name of Dr. Long by awarding him a place in the Hall of Fame in Washington, D. C., but the niche is yet unfilled.

Let every physician consider this a personal matter and one in which his pride is at stake. A worthy monument will do much to dispel further disputes as to who had priority in the discovery of ether and will leave a record for the enlightenment of coming generations.

Dr. Hardman has agreed to erect a monument to Long at the original home of Long and the place in which ether was first administered.

